# Identification of dominant gas transport frequencies during barometric pumping of fractured rock

**DOI:** 10.1038/s41598-019-46023-z

**Published:** 2019-07-02

**Authors:** Dylan R. Harp, John P. Ortiz, Philip H. Stauffer

**Affiliations:** Computational Earth Science, Los Alamos National Laboratory, Los Alamos, NM 87544 USA

**Keywords:** Environmental impact, Hydrology

## Abstract

We demonstrate that although barometric pressures are complicated signals comprised of numerous frequencies, it is a subset of these frequencies that drive the overwhelming majority of gas transport in fractured rock. Using an inverse numerical analysis, we demonstrate that a single barometric component with seasonally modulated amplitude approximates gas transport due to a measured barometric signal. If past barometric tendencies are expected to continue at a location, the identification of this frequency can facilitate accurate long term predictions of barometrically induced gas transport negating the need to consider stochastic realizations of future barometric variations. Additionally, we perform an analytical analysis that indicates that there is a set of barometric frequencies, consistent with the inverse numerical analysis, with high production efficiency. Based on the corroborating inverse numerical and analytical analyses, we conclude that there is a set of dominant gas transport frequencies in barometric records.

## Introduction

**Figure 1:**
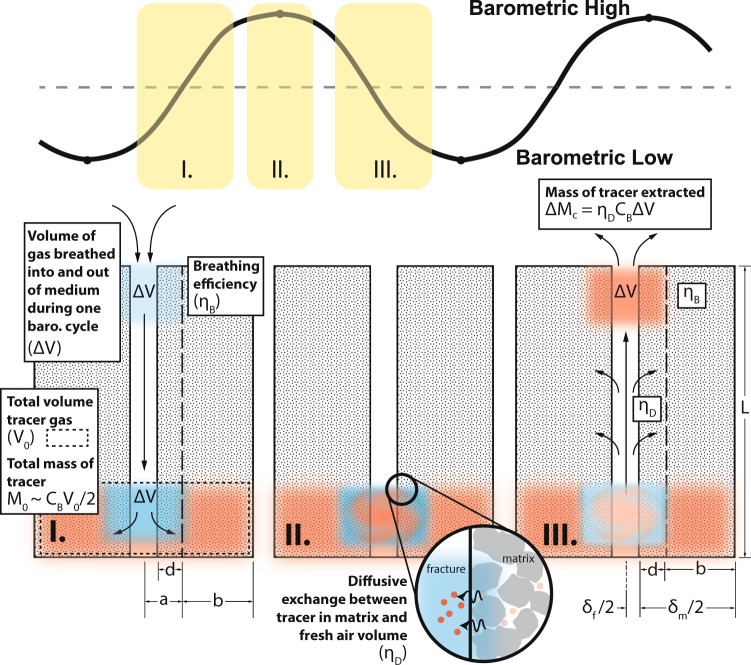
Schematic representation of barometric pumping of gas through a fracture. The portion of the barometric curve that Panel I, II, III are associated with is indicated along the top of the schematic. The breathing efficiency (*η*_*B*_), diffusive exchange efficiency (*η*_*D*_), and important quantities used and defined in the analytical analysis are indicated. *C*_*B*_ is the tracer concentration.

Predictions of gas transport can be improved for many applications by better understanding the effect of barometric variations on gas transport in fractured rock. Barometric variations push gases deeper into the fractured rock during barometric highs and pull gases upward during barometric lows^1–5^. Figure 1 contains a schematic conceptualization of this process with details that will be referred to throughout this paper. Fractures provide fast pathways for gas transport while the rock matrix provides relatively immobile gas storage in between barometric cycles, allowing for a ratcheting mechanism that greatly enhances gas transport. Barometric variations can drive leakage from CO_2_ sequestration sites^6–10^, leakage of methane from hydraulic fracturing operations^11^, radon gas entry into buildings^12^, and radionuclide gas seepage from underground nuclear explosions and waste storage^13–19^.

One of the primary uncertainties in making predictions of barometrically-induced gas transport is that the future barometric variations are highly uncertain and cannot be accurately forecasted past a few weeks. Barometric pressure fluctuations are complex signals composed of many frequencies driven by multiple processes including atmospheric tides, weather patterns, and seasonal and annual cycles and are highly dependent on latitude and elevation. Many previous researchers have assumed that barometric variations due to roughly weekly weather patterns are important frequencies to consider for gas transport in fractured rock. Nilson *et al*.^1^ identified that the barometric period with the highest amplitude at the National Nuclear Security Site (NNSS) was approximately 7.2 days based on historical data. Mourzenko *et al*.^20^ use a synthetic sinusoidal barometric signal with period of approximately 7.3 days to represent weather patterns at Roselend Natural Laboratory in France for their numerical investigations. Neeper^3^ also assumed that weekly weather patterns are important for gas transport in fractured rock. However, in our investigations, a spectral analysis of a barometric record results in many frequencies with similar amplitude making it difficult to identify a single dominant barometric frequency. We also demonstrate that the barometric sinusoidal component with the largest amplitude will not necessarily be associated with the dominant gas transport frequency.

This paper presents the first detailed analysis of the identification of the dominant gas transport frequency through corroborating inverse numerical and analytical analyses. Using barometric pressures measured in Anchorage, AK from 2014 through 2017, we first present the decomposition of a barometric pressure record into sinusoidal components (amplitude/frequency pairs). Anchorage was chosen since it is representative of a location with large barometric variations. Then, we present an inverse analysis using a numerical model of gas transport within a fractured domain demonstrating that a dominant transport frequency can be identified that reproduces gas transport simulated using the measured barometric pressures. Next, we perform an analytical analysis of gas production efficiency using the decomposed barometric pressure record. From this we identify a set of highly efficient frequencies for producing gas that are clustered around the frequency identified in the inverse numerical analysis. Finally, we provide conclusions based on the corroborating evidence from the inverse numerical and analytical analyses.

## Barometric Pressure Decomposition

**Figure 2:**
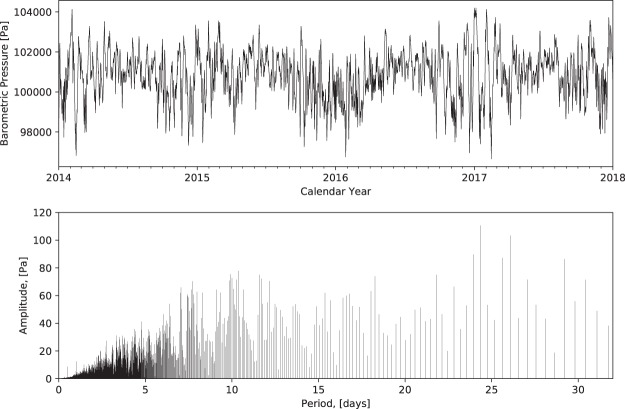
Measured barometric pressure record from Anchorage, AK (top) and associated amplitude/period pairs (bottom).

We obtained hourly barometric pressure data from Anchorage, AK from 2014 through 2017 from Weather Underground (www.wunderground.com). In order to obtain a uniform one hour spacing, we linearly interpolated a few missing measurements and removed a few extra intra-hour measurements in the record (top plot in Fig. 2). We decomposed the data into the frequency domain using a Fast Fourier Transform (FFT) algorithm^21^ in the bottom plot of Fig. 2, where the barometric period is *T* = 2*π*/*ω*, where *ω* is the barometric frequency. From this plot, it is apparent that the barometric signal is a complicated combination of many components (frequency/amplitude pairs). While a period of around 24 days has the largest amplitude, our analysis below will demonstrate that this is not the dominant gas transport frequency. Therefore, unlike the case in Nilson *et al*.^1^, where the dominant gas transport frequency is identified as the frequency with the largest amplitude, it is not possible to identify the dominant gas transport frequency simply based a spectral analysis alone.

## Numerical Modeling Analysis

We use a 2D numerical model to simulate air flow and gas transport and immobile pore-water storage of dissolved gas within a partially-saturated, fractured rock with 1 mm vertical fractures separated by 10 m. The 2D planar model domain comprises a vertical half fracture (*δ*_*f*_/2 = 0.5 mm) connected to a matrix half block (*δ*_*m*_/2 = 5 m) extending 100 m deep (refer to Panel III of Fig. 1). The vertical sides of the model are reflection (zero-flux Neumann) boundaries allowing the half fracture/half matrix block to represent a series of fractures/matrix blocks. The bottom is also a zero-flux Neumann boundary representing an extremely low permeability bedrock or a water table. The bottom 5 m of the model is populated with a gas tracer concentration of 1 mol/L. A time-varying pressure boundary is applied to the top of the model representing barometric variations. The model is implemented in the PFLOTRAN simulator^22^ with modifications to allow for kinetic dissolution and exsolution of gas between air and immobile pore-water. The model is described in detail and verified against a suite of analytical solutions in Harp *et al*.^5^.

### Identifying the dominant gas transport frequency

**Figure 3:**
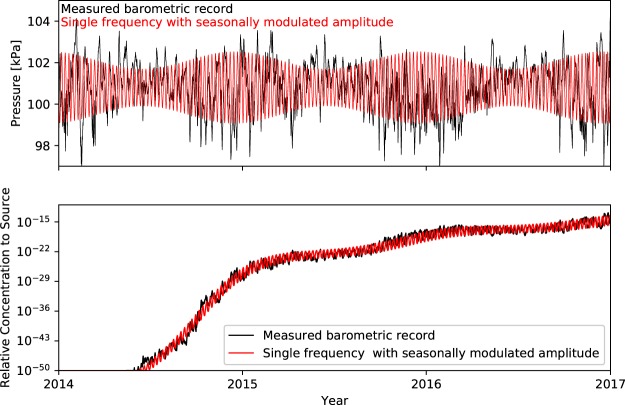
Top: Calibrated barometric signal with single, constant amplitude frequency (red line) with the measured barometric record (black line) for reference. Bottom: Gas concentrations (relative to the source concentration) at the top of the fracture (ground surface) simulated using a calibrated single frequency barometric signal with seasonally modulated amplitude from the top plot (red line). The relative concentrations driven by the measured barometric signal from the top plot (black line; calibration targets) are provided for reference.

We calibrate the synthetic pressures using a Levenberg-Marquardt optimization approach^23^ implemented in the PEST software package^24^. An initial calibration using a single frequency synthetic barometric signal with constant amplitude resulted in a seasonal mismatch in concentrations (for details, refer to Section S1 in Supporting Material). By inspecting the top plot in Fig. 2, it is apparent that the barometric pressures during the summer months have lower amplitudes than during winter months. We therefore defined a seasonally modulated synthetic barometric pressure signal as

1 $${P}_{s}(\theta )=({{\mathscr{A}}}_{d}+{{\mathscr{A}}}_{s}\,\sin ({\omega }_{s}t+{\gamma }_{s}))\sin ({\omega }_{d}t+{\gamma }_{d}),$$

where ${{\mathscr{A}}}_{d}$ is the mean amplitude of the dominant gas transport frequency, ${{\mathscr{A}}}_{s}$ is the amplitude of the seasonal modulation, *ω*_*d*_ is the dominant gas transport frequency, *ω*_*s*_ is the seasonal modulation frequency (*T*_*s*_ = 1 year, where *ω*_*s*_ = 2*π*/*T*_*s*_), and *γ*_*d*_ and *γ*_*s*_ are the phase shift of the dominant gas transport frequency and seasonal modulation, respectively, and $\theta =[{{\mathscr{A}}}_{d},{T}_{d},{\gamma }_{d},{{\mathscr{A}}}_{s},{\gamma }_{s}]$ is a vector containing the calibration parameters. The (${{\mathscr{A}}}_{d}+{{\mathscr{A}}}_{s}\,\sin ({\omega }_{s}t+{\gamma }_{s})$) term captures the seasonal modulation about the mean of the dominant gas transport frequency. The objective function *F* minimized in the calibration is

2 $$F(\theta )=\sum _{i=1}^{N}({C}_{i}^{m}-{C}_{i}^{s}(\theta )),$$

where ${C}_{i}^{m}$ and ${C}_{i}^{s}$ are the *i*th tracer concentrations at the ground surface driven by the measured and synthetic barometric signals, respectively. We plot the calibrated seasonally modulated barometric signal in the top plot of Fig. 3 as the red line along with the measured barometric record as the black line. In the bottom plot of Fig. 3, concentrations simulated using the synthetic barometric signal (red line) and measured barometric record (black line) are plotted, demonstrating that a seasonally modulated single frequency barometric signal is able to closely approximate concentrations driven by a measured barometric record. This calibration reduced the standard error of the concentration residuals by approximately half compared to the calibration with constant amplitude barometric frequency (Section S1 in Supporting Material).

The calibrated parameter values are ${{\mathscr{A}}}_{d}=1310$ Pa, *T*_*d*_ = 7.29 days, ${{\mathscr{A}}}_{s}=422$ Pa, and *γ*_*s*_ = 1.85 radians. We calibrated *γ*_*d*_, but the calibration was insensitive to its value because the gas transport in our example is a longer term process resulting from a succession of barometric cycles, and therefore identifying a phase shift is not critical to match the overall concentration trend.

### Sensitivity of calibrated barometric parameters to subsurface domain scenarios

Based on an analysis of the sensitivity of tracer concentrations at the top of the fracture to subsurface domain parameters, we identified that the concentrations are most sensitive to the depth to impermeable layer *L* and matrix permeability *k*_*m*_ (refer to Section S2 in Supporting Material for details). Therefore, since *L* and *k*_*m*_ have the largest potential to alter the calibration of the barometric parameters (${{\mathscr{A}}}_{d},{T}_{d},{{\mathscr{A}}}_{s},{\gamma }_{s}$), we focus on these subsurface domain parameters for further investigation.

We perform a joint inversion to calibrate the barometric parameters to match the concentrations simulated using the measured barometric record from 4 subsurface domain scenarios: (1) *L* = 50 m; (2) *L* = 150 m; (3) *k*_*m*_ = 10^−19^ m^2^, and (4) *k*_*m*_ = 10^−17^ m^2^, with the other subsurface properties at their base case values (*δ*_*m*_ = 10 m, *L* = 100 m, matrix porosity *ϕ*_*m*_ = 0.01 m^3^/m^3^, *k*_*m*_ = 10^−18^ m^2^, *δ*_*f*_ = 1 mm, and matrix saturation *S*_*m*_ = 0.5 m^3^/m^3^). The objective function for this joint inversion can be described by extending Eq. 2 as

3 $$F(\theta )=\sum _{j=1}^{4}\,\sum _{i=1}^{{N}_{j}}\,({C}_{ij}^{m}-{C}_{ij}^{s}(\theta )),$$

where ${C}_{ij}^{m}$ and ${C}_{ij}^{s}$ are the *i*th concentrations of the *j*th simulation driven by the measured and synthetic barometric signal, respectively, with $\theta =[{{\mathscr{A}}}_{d},{T}_{d},{{\mathscr{A}}}_{s},{\gamma }_{s}]$ in this case.

**Figure 4:**
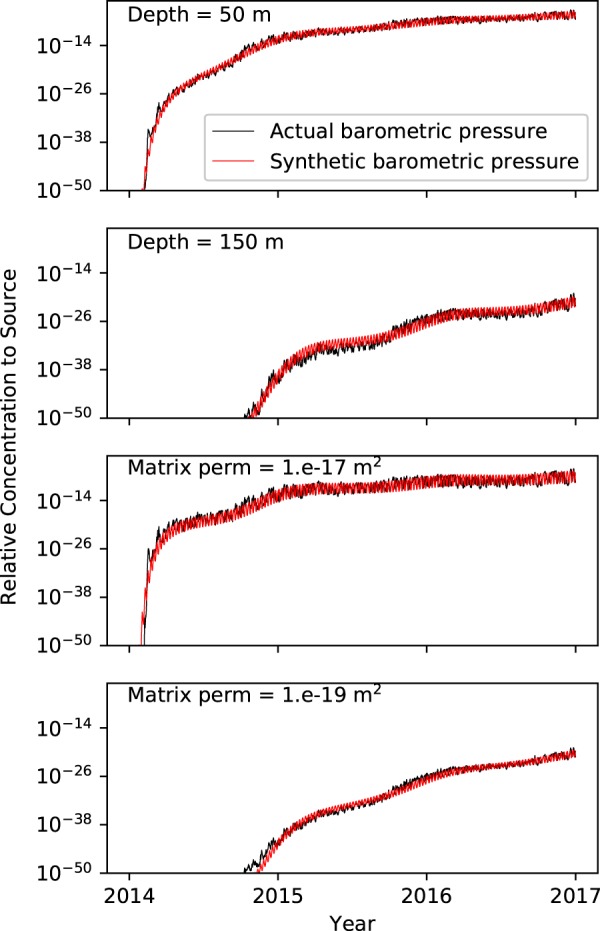
Results of a joint calibration of a seasonally-modulated single frequency barometric signal including concentrations from 4 subsurface domain scenarios. Concentrations at the top of the fracture (ground surface) driven by measured barometric pressures (calibration targets) are black lines and calibrated concentrations are red lines.

In Fig. 4, we present the calibrated concentrations (red lines) against the concentrations simulated with measured barometric pressures (black lines). These plots illustrate that it is possible to use a single synthetic barometric signal with seasonally modulated amplitude to capture concentrations simulated with an actual barometric signal for various subsurface model domains and properties. The calibrated parameter values from this inversion are ${{\mathscr{A}}}_{d}=1326$ Pa, *T*_*d*_ = 7.29 days, ${{\mathscr{A}}}_{s}=419$ Pa, *γ*_*s*_ = 1.59 radians. These values are similar to those from the base case inversion presented above.

## Analytical Analysis

The *mass discharge efficiency* of a barometric frequency is dependent on its ability to push and retract atmospheric air into and out of fractured rock and allow sufficient time for the diffusive exchange of gas into this air while at the depth of the tracer, and retain the gas during the return trip to the atmosphere (i.e., not lose too much gas due to diffusion into matrix without tracer). The *mass discharge efficiency* (*η*_*M*_) can be defined as the mass of tracer removed from the subsurface during one barometric cycle (Δ*M*_*c*_) relative to the original mass of tracer in the subsurface (*M*_0_) as

4 $${\eta }_{M}=\frac{{\rm{\Delta }}{M}_{c}}{{M}_{0}},$$

where, assuming a linear concentration gradient with depth, *M*_0_ can be approximated as

5 $${M}_{0}={C}_{B}{V}_{0}/\mathrm{2,}$$

where *C*_*B*_ is the concentration of the tracer gas and *V*_0_ is the volume of air with tracer^1^. The *dominant gas transport frequency* will not only depend on *η*_*M*_ but will also depend on the frequency of occurrence of the barometric component. For example, a barometric component with lower *η*_*M*_ may remove more gas from the subsurface over time than a less frequent component with higher *η*_*M*_ simply because it occurs more often. We define the *production efficiency* (*η*_*P*_) as a metric for identifying *dominant gas transport frequencies* by multiplying *η*_*M*_ by the frequency as

6 $${\eta }_{P}=\frac{{\rm{\Delta }}{M}_{c}}{{M}_{0}}\omega ={\eta }_{M}\omega \mathrm{.}$$

In order to approximate Δ*M*_*c*_ analytically, the exchange of fresh air with air containing tracer can be conceptualized as occurring within a packet of air with volume Δ*V* that travels down the fracture and a portion of the matrix wall that has been invaded by incoming fresh air^1^. Figure 1 illustrates this conceptualization and many of the quantities used below. The thickness of this packet of air in half-space considering both the half fracture thickness and the invaded region of the associated matrix half block (*d*) is $a=\frac{{\delta }_{f}}{2}+d$. The remainder of the matrix half block is then the region with tracer which has a thickness of $b=\frac{{\delta }_{m}}{2}-d$. Based on this conceptualization, Nilson *et al*.^1^ derive an analytical equation for Δ*M*_*c*_ (refer to Panel III of Fig. 1) as

7 $${\rm{\Delta }}{M}_{c}=-\frac{\pi }{4}{C}_{B}{\rm{\Delta }}V\,Re\,[\frac{i{W}_{a}\sqrt{i}}{{W}_{a}\sqrt{i}+\,\tanh (\beta {W}_{a}\sqrt{i})}],$$

where *W*_*a*_ is the Womersley number modified to account for the porosities of the given problem defined as ${W}_{a}=\frac{{\varphi }_{c}a}{{\varphi }_{m}}\sqrt{\frac{\omega }{D}}$ and *β* is the ratio of the volume of clean air versus air with tracer calculated as $\beta =\frac{b{\varphi }_{m}}{a{\varphi }_{c}}$, where *ϕ*_*c*_ is calculated as the volume weighted average porosity of the fracture and invaded region as ${\varphi }_{c}=\frac{{\varphi }_{m}d+{\varphi }_{f}{\delta }_{f}/2}{d+{\delta }_{f}/2}$, where *ϕ*_*f*_ is one in our case, and $i=\sqrt{-1}$.

If the subsurface were in perfect equilibrium with the surface, i.e., the pneumatic diffusivity were extremely large so that pressure variations at the ground surface are immediately transferred throughout the subsurface, the ratio of Δ*V* to the total volume of subsurface air with tracer *V*_0_ would be proportional to the ratio of the change in pressure (amplitude) Δ*p* to the mean static pressure *p*_0_ as

8 $$\frac{{\rm{\Delta }}V}{{V}_{0}}=\frac{{\rm{\Delta }}p}{{p}_{0}}\mathrm{.}$$

Based on this relationship, the conceptual maximum volume of air that could be extracted from the subsurface due to a single cycle of a barometric component is

9 $${\rm{\Delta }}{V}_{\max }=\frac{{\rm{\Delta }}p}{{p}_{0}}L({\delta }_{f}+{\varphi }_{m}{\delta }_{m}),$$

where the variables have been defined earlier. However, due to finite pneumatic diffusivity in the fracture and matrix, perfect pressure equilibrium is never achieved throughout the subsurface. Using a double porosity (fracture/matrix) analytical solution, Nilson *et al*.^1^ derive an analytical solution for Δ*V* as

10 $${\rm{\Delta }}V=\frac{{\rm{\Delta }}p}{{p}_{0}}L{\delta }_{f}\,{\rm{mod}}\,[\frac{{\lambda }_{fm}^{2}}{{\lambda }_{f}^{2}}\frac{\tanh \,{\lambda }_{fm}\sqrt{i}}{{\lambda }_{fm}\sqrt{i}}],$$

where the *λ*’s are dimensionless Fourier numbers that define the ratio of the diffusive transport rate to the storage rate defined as

11 $${\lambda }_{fm}={\lambda }_{f}(1+\frac{{\varphi }_{m}{\delta }_{m}}{{\delta }_{f}}\frac{\tanh \,{\lambda }_{m}\sqrt{i}}{{\lambda }_{m}\sqrt{i}}),$$

12 $${\lambda }_{f}=L\sqrt{\omega /{\alpha }_{f}}$$

and

13 $${\lambda }_{m}={\delta }_{m}/2\sqrt{\omega /{\alpha }_{m}},$$

where the subscripts *fm*, *f*, and *m* denote the composite fracture and matrix, fracture only, and matrix only, respectively. *α*_*f*_ and *α*_*m*_ are pneumatic diffusivities, where *α*_*f*_ is defined using the cubic law as ${\alpha }_{f}={\delta }_{f}^{2}{p}_{0}/(12\mu )$ and *α*_*m*_ is *α*_*m*_ = *k*_*m*_*p*_0_/(*ϕ*_*m*_*μ*), where *μ* is air viscosity.

Inserting Eq. 10 into Eq. 7, and then Eqs 5 and 7 into Eq. 6, *η*_*P*_ can be expressed as

14 $${\eta }_{P}=\frac{{\rm{\Delta }}{M}_{c}}{{M}_{0}}\omega =-\frac{\pi {\rm{\Delta }}pL{\delta }_{f}\omega }{2{V}_{0}{p}_{0}}\,{\rm{mod}}\,[\frac{{\lambda }_{fm}^{2}}{{\lambda }_{f}^{2}}\frac{\tanh \,{\lambda }_{fm}\sqrt{i}}{{\lambda }_{fm}\sqrt{i}}]\,{\rm{Re}}\,[\frac{i{W}_{a}\sqrt{i}}{{W}_{a}\sqrt{i}+\,\tanh (\beta {W}_{a}\sqrt{i})}].$$

This equation can also be derived from the *breathing efficiency* (*η*_*B*_), which quantifies the volume of air that a barometric cycle is able to extract (Δ*V*) relative to the maximum volume that could be removed if the subsurface were in perfect equilibrium with the atmosphere (Δ*V*_*max*_), defined as

15 $${\eta }_{B}=\frac{{\rm{\Delta }}V}{{\rm{\Delta }}{V}_{\max }}=\frac{{\delta }_{f}}{{\delta }_{f}+{\varphi }_{m}{\delta }_{m}}\,{\rm{mod}}\,[\frac{{\lambda }_{fm}^{2}}{{\lambda }_{f}^{2}}\frac{\tanh \,{\lambda }_{fm}\sqrt{i}}{{\lambda }_{fm}\sqrt{i}}],$$

and the *diffusive exchange efficiency* (*η*_*D*_), which quantifies the fraction of the mass of air with tracer that is removed (Δ*M*_*c*_) versus the maximum mass that would be removed if the concentration of the packet of air could achieve and maintain the concentration of the air with tracer in the subsurface (*C*_*B*_Δ*V*), defined as

16 $${\eta }_{D}=\frac{{\rm{\Delta }}{M}_{c}}{{C}_{B}{\rm{\Delta }}V}=-\,\frac{\pi }{4}\,{\rm{Re}}\,[\frac{i{W}_{a}\sqrt{i}}{{W}_{a}\sqrt{i}+\,\tanh (\beta {W}_{a}\sqrt{i})}].$$

Refer to Fig. 1 for depictions of *η*_*B*_ and *η*_*D*_. Nilson *et al*.^1^ combine *η*_*B*_ and *η*_*D*_ into the *overall transport efficiency η* as

17 $$\eta ={\eta }_{B}{\eta }_{D}$$

to describe the efficiency of a single cycle of a barometric component to extract gas from the subsurface (i.e., considering a single cycle in isolation, not the ability of the barometric component to extract gas from the subsurface per unit of time). As such, *production efficiency* can be equivalently expressed as

18 $${\eta }_{P}={\eta }_{B}{\eta }_{D}\omega =\eta \omega .$$

**Figure 5:**
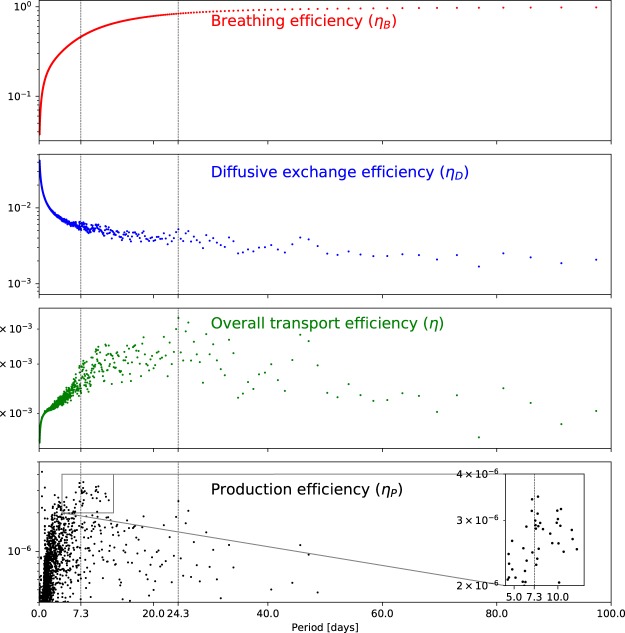
Breathing, diffusive exchange, overall transport, and production efficiencies for the amplitude/frequency pairs of the measured barometric record plotted as function of barometric period.

In Fig. 5, we plot the breathing efficiency (*η*_*B*_), diffusive exchange efficiency (*η*_*D*_), overall transport efficiency (*η*), and production efficiency (*η*_*P*_) calculated for the components of the measured barometric record as a function of period. In the top plot, it is apparent that *η*_*B*_ increases monotonically with barometric period (decreases with frequency). It is not dependent on amplitude, which cancels out during its derivation. Therefore, *η*_*B*_ effectively quantifies the ability of lower frequency components to more effectively penetrate into fractured rock irrespective of their amplitude. In general, *η*_*D*_ decreases with increasing period, however, the combination of period and amplitude leads to more nuanced (non-monotonic) behavior. The combination of these conflicting efficiencies in *η* leads to a maximum efficiency at around 24.3 days (refer to the vertical dashed line in Fig. 5). Referring to the bottom plot of Fig. 2, this is the period with the largest amplitude. As discussed previously, *η* quantifies the gas transport efficiency of a single cycle of a barometric component and does not account for the frequency of occurrence of the barometric component. Therefore, it is not appropriate for identifying the dominant gas transport barometric component.

**Figure 6:**
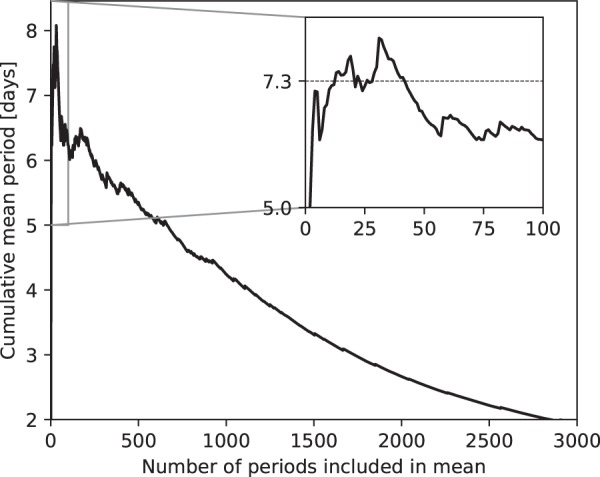
Cumulative mean period for amplitude/frequency pairs of the measured barometric record sorted in order of decreasing production efficiency.

In the bottom plot of Fig. 5, we plot the production efficiency (*η*_*P*_; Eq. 14) as a function of barometric period. In this case, when the frequency of occurrence of the barometric component is taken into account, it is apparent that there is a cluster of high efficiency amplitude/frequency pairs around a period of 7.3 days (refer to the inset in Fig. 5), consistent with the inverse numerical analysis above. However, the results are complicated by the highest production efficiency occurring at 0.5 days. To investigate this, in Fig. 6, we plot the cumulative mean period of the amplitude/frequency pairs sorted in order of decreasing production efficiency. This demonstrates that although the highest production efficiency occurs at 0.5 days, the average period of the highest efficiency amplitude/frequency pairs hovers around 7.3 days. Therefore, it is likely that for the Anchorage, AK data that we analyzed, 7.3 days is the average period of a set of high production efficiency periods that lead to the vast majority of the gas transport.

## Conclusions

The combined inverse numerical and analytical analyses presented in this manuscript support the following conclusions:

1. There is a set of barometric frequencies responsible for the vast majority of gas transport in fractured rock.
2. A single barometric frequency with seasonally modulated amplitude can be used to accurately predict the barometrically-induced gas transport from a measured barometric signal.
3. The dominant gas transport frequency is the average of the high production efficiency amplitude/frequency barometric sinusoidal components.

For practical applications, these conclusions indicate that as long as future barometric pressures at a location have similar characteristics to the past, it is possible to predict future gas transport using a single seasonally modulated barometric frequency. This eliminates the need to consider an ensemble of possible future barometric variations, significantly simplifying predictions of gas transport in fracture rock and subsequent breakthrough times.

## Supplementary information

Supporting Material

## Data Availability

The data used in this research is freely available from Weather Underground (www.wunderground.com).
