# Supplementary material for: Identification of dominant gas transport frequencies during barometric pumping of fractured rock
Source: Sci Rep. 2019 Jul 2;9:9537. doi: 10.1038/s41598-019-46023-z (PMC6606586; doi:10.1038/s41598-019-46023-z)
Supplement: Supplementary file 1 — Supporting Material [file 41598_2019_46023_MOESM1_ESM.pdf]

Support material for ‘Identification of dominant gas transport frequencies during barometric pumping of fractured rock’

Dylan R. Harp[1,*], John P. Ortiz[1], and Philip H. Stauffer[1]

[1]Computational Earth Science, Los Alamos National Laboratory, Los Alamos, NM, 87544, USA
[*]dharp@lanl.gov

S1 Calibration with constant amplitude barometric frequency

Here, we test whether gas transport driven by a complicated, real barometric signal can be approximated by that driven by a synthetic barometric signal with a single constant amplitude frequency. We accomplish this by calibrating the synthetic barometric signal to match surface concentrations simulated with the measured barometric record. The single frequency barometric signal is defined as

$$P_d(\theta) = \mathscr{A}_d \sin\left(\frac{2\pi}{T_d}t + \gamma_d\right) = \mathscr{A}_d \sin(\omega_d t + \gamma_d) \tag{S1}$$

where $\mathscr{A}_d$ is the synthetic amplitude, T_d is the synthetic period, ω_d is the synthetic frequency, where $T_d = 2\pi/\omega_d$, and γ_d is the phase shift. We calibrate the synthetic pressures using a Levenberg-Marquardt optimization approach[1] implemented in the PEST software package[2]. The objective function F minimized in the calibration is

$$F(\theta) = \sum_{i=1}^{N}(C_i^m - C_i^s(\theta)) \tag{S2}$$

where $\theta = [\mathscr{A}_d, T_d, \gamma_d]$ is a vector containing the calibration parameters.

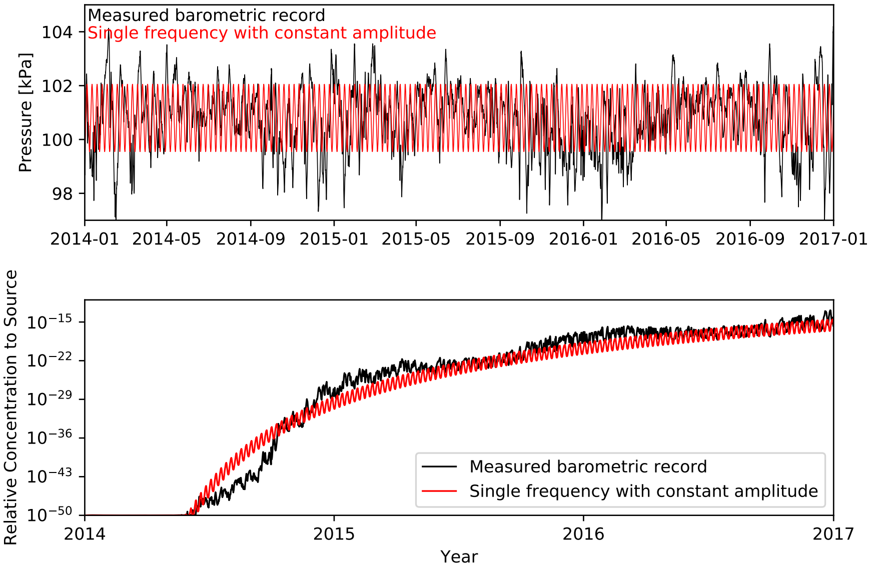

Figure S1. Top: Calibrated barometric signal with single, constant amplitude frequency (red line) with the measured barometric record (black line) for reference. Bottom: Gas concentrations (relative to the source concentration) at the top of the fracture (ground surface) simulated using a calibrated single frequency barometric signal with constant amplitude from the top plot (red line). The relative concentrations driven by the measured barometric signal from the top plot (black line; calibration targets) are provided for reference.

S2 Subsurface domain scenario sensitivity analysis

To identify the subsurface parameters that the gas transport is most sensitive to, we simulate concentrations driven by the measured barometric record for values of fracture spacing (matrix block width $\delta_m : \{5, 15\}$ m), depth to the impermeable layer ($L : \{50, 150\}$ m), matrix porosity ($\phi_m : \{0.005, 0.015\}$ m^3/m^3), matrix permeability ($k_m : \{10^{-19}, 10^{-17}\}$ m^2), fracture width ($\delta_f : \{0.5, 1.5\}$ mm), and matrix saturation ($S_m : \{0.25, 0.75\}$ m^3/m^3) around their base case values ($\delta_m = 10$ m, $L = 100$ m, $\phi_m = 0.01$ m^3/m^3, $k_m = 10^{-18}$ m^2, $\delta_f = 1$ mm, and $S_m = 0.5$ m^3/m^3). By inspecting the deviation in concentrations for these parameter values in Figure S2, the concentrations are most sensitive to the depth L and matrix permeability k_m, so we focus on these parameters for further investigation since they have the largest potential to alter the calibration of the barometric parameters ($\mathscr{A}_d, T_d, \mathscr{A}_s, \gamma_s$).

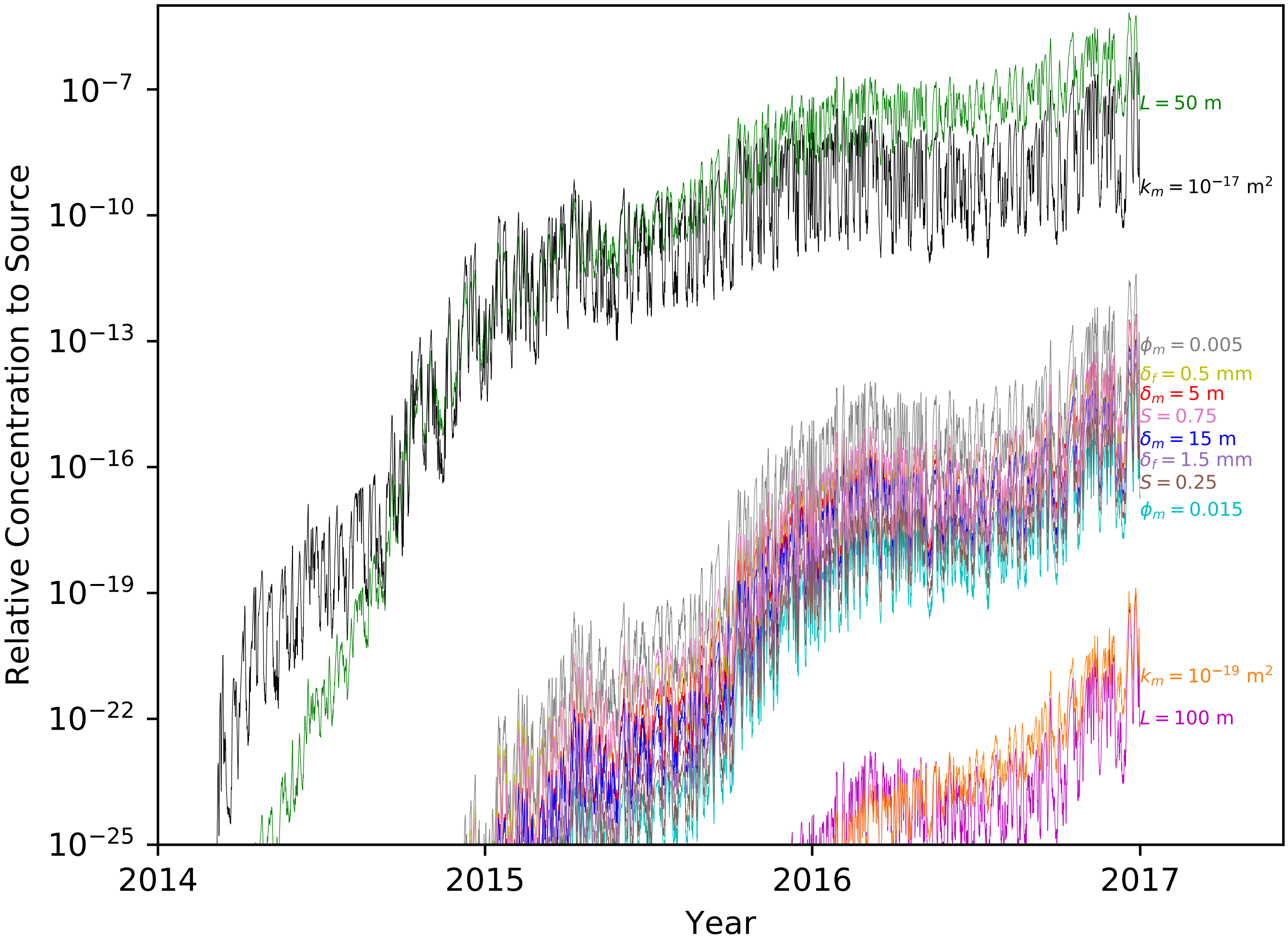

Figure S2. Sensitivity analysis of gas transport (relative concentration to source at the ground surface) to subsurface domain parameters.

References

1. Marquardt, D. W. An algorithm for least-squares estimation of nonlinear parameters. *J. society for Ind. Appl. Math.* **11**, 431–441 (1963).

2. Doherty, J. PEST-ASP user's manual. *Watermark Numer. Comput. Brisbane, Aust.* (2001).
